# Deep Learning and Neural Architecture Search for Optimizing Binary Neural Network Image Super Resolution

**DOI:** 10.3390/biomimetics9060369

**Published:** 2024-06-18

**Authors:** Yuanxin Su, Li-minn Ang, Kah Phooi Seng, Jeremy Smith

**Affiliations:** 1 XJTLU Entrepreneur College (Taicang), Xi’an Jiaotong Liverpool University, Taicang 215400, China; 2 Department of Electrical Engineering and Electronics, University of Liverpool, Liverpool L69 3GJ, UK; 3 School of Science, Technology and Engineering, University of the Sunshine Coast, Moreton Bay, QLD 4502, Australia

**Keywords:** deep learning, neural architecture search, binary neural network, image super resolution

## Abstract

The evolution of super-resolution (SR) technology has seen significant advancements through the adoption of deep learning methods. However, the deployment of such models by resource-constrained devices necessitates models that not only perform efficiently, but also conserve computational resources. Binary neural networks (BNNs) offer a promising solution by minimizing the data precision to binary levels, thus reducing the computational complexity and memory requirements. However, for BNNs, an effective architecture is essential due to their inherent limitations in representing information. Designing such architectures traditionally requires extensive computational resources and time. With the advancement in neural architecture search (NAS), differentiable NAS has emerged as an attractive solution for efficiently crafting network structures. In this paper, we introduce a novel and efficient binary network search method tailored for image super-resolution tasks. We adapt the search space specifically for super resolution to ensure it is optimally suited for the requirements of such tasks. Furthermore, we incorporate Libra Parameter Binarization (Libra-PB) to maximize information retention during forward propagation. Our experimental results demonstrate that the network structures generated by our method require only a third of the parameters, compared to conventional methods, and yet deliver comparable performance.

## 1. Introduction

Super resolution (SR) is an essential task in computer vision, aimed at designing effective models to reconstruct high-resolution (HR) images from low-resolution (LR) ones. It plays a vital role across various domains, such as medical imaging [1,2], biometric information identification [3,4,5], and astronomical images [6].

Traditional SR interpolation methods [7] offer rapid processing, but fall short in terms of accuracy. The field has thus evolved from these methods to adopting deep learning techniques, such as [8,9,10,11,12], which provide enhanced performance. Nevertheless, the growing complexity and escalated computational demands of deep neural networks make it difficult to deploy these models on devices with limited resources. As network architecture search has advanced, the introduction of DARTS [13] has significantly lowered the barrier to entry for this technology. This development makes differentiable NAS an attractive option for achieving lightweight super resolution (SR), particularly because manually designing lightweight methods often involves considerable time spent on trial and error. DARTS can relieve designers from the cumbersome process of manual design. This method has enabled efficient searches for neural network architectures within a continuous search space. DARTS models neural architecture search (NAS) as a bi-level optimization problem, employing alternate optimization through gradient descent to derive the optimal network architecture. DARTS is also categorized under one-shot NAS methods, which involve constructing a super net and then extracting the best sub-network from it. This approach addresses the traditional black box nature of network structure searches, making them more transparent and detailed. The key lies in its use of soft functions to mix candidate operations. The gradient optimization approach pioneered by DARTS demonstrates remarkable results, evident from the minimal GPU days required to see outcomes [14]. This method stands out from other early NAS techniques [15,16,17], particularly those based on reinforcement learning or evolutionary algorithms, because it is not constrained by the intrinsic discrete optimization nature of these methods. Fundamentally, it addresses the issue that previous NAS methods required extensive computational resources.

For lightweight models, an optimal network structure is just one part of the equation. Introducing specific data types can also make networks more efficient and lightweight. Binary neural networks (BNNs), which map full-precision data to binary values {−1, 1} [18], are particularly hardware friendly. This attribute not only speeds up processing, but also decreases memory pressure, significantly easing the load on hardware resources during model computation. However, at present, most research on binary networks focuses on adaptations of established full-precision architectures, including ResNet [19] and DenseNet [20]. Notable examples include Bi-Real net [21], binary DenseNet [22], and the Binarized Ghost Module (BGM) [23]. Although Bethge et al. [22] proposed architectural design principles through extensive experimentation and demonstrated their effectiveness in creating efficient new architectures, manually designing an appropriate architecture remains a task that rapidly depletes both resources and time. To address the complexities of designing the architecture of BNNs, researchers have begun to explore the application of NAS in BNNs. Nevertheless, nowadays, most of the existing work on NAS only focuses on real-valued architecture design. Using real-valued NAS strategies in binary domains often results in inadequate convergence. Prior research has validated this issue, highlighting the following principal reasons behind it:

1. An unsuitable search space for BNNs;
2. Conducting architecture searches with binary weights and activations can result in topological degeneration or the training merely converging to an extremely low accuracy;
3. Mismatches between the search process and evaluation;
4. The imbalanced selection of operations.

Although they each proposed solutions designed to enhance model performance in image classification, these methods might not be equally effective for super-resolution tasks and could potentially degrade the performance. Therefore, we have revised the original solutions to better suit the search for optimal binary network structures specifically for super-resolution tasks.

In summary, our main contributions include the following:

1. We define a friendly search space for BNNs for the task of super resolution;
2. We further stabilize the search process by applying L1 and L2 regularization, making the selection of operations more equitable during the search process. Additionally, we explore the effects of different combinations of L1 and L2 regularization;
3. We modify the basic architecture of the model to preserve image information through a hierarchical basic architecture, as much as possible;
4. Given the unique demands of super-resolution (SR) tasks, which differ from image classification in that SR models need to preserve information to the greatest extent possible, we introduce Libra Parameter Binarization (Libra-PB) [24] to maximize the retention of information during forward propagation. For backward propagation, the introduction of the Error Decay Estimator (EDE) helps the model effectively manage the loss of information caused by the reduced parameter update capability outside the truncation range and the approximate errors within it. These measures are designed to minimize the mismatch between the search and evaluation;
5. Our approach is capable of generating superior BNN architectures for super-resolution tasks, with relatively low computational expenditure.

The paper is structured as follows. The earlier sections provide background information on the domain areas of the research. Section 2 presents the discussions on image super resolution, Section 3 presents the discussions on binary neural networks, and Section 4 presents the discussions on neural architecture search. Section 5 discusses the methodology used in the paper to develop a deep learning and neural architecture search that is targeted towards optimizing binary neural network image super-resolution tasks. Section 6 discusses the experimental results to validate the proposed approaches. This section also provides comparisons with other approaches to image super resolution (SR). Some concluding remarks are given in Section 7.

## 2. Image Super Resolution

The SRCNN [8] marks the pioneering work of deep learning in super-resolution reconstruction. The network structure of SRCNN is very simple, utilizing just three convolutional layers. The network architecture is illustrated in the figure below. SRCNN first enlarges the low-resolution image to the target size using bicubic interpolation, then it applies a three-layer convolutional network to approximate the nonlinear mapping, and finally outputs the high-resolution image result. In this paper, the author interprets the three-layer convolutional structure as three steps: the extraction and feature representation of the image blocks, the nonlinear mapping of the features, and the final reconstruction.

The FSRCNN [25] and SRCNN are works by Dong Chao, Xiaoou Tang, and others from the Chinese University of Hong Kong. FSRCNN is an improvement on the previous SRCNN in regard to three main aspects. First, it uses a deconvolution layer at the end to scale up the size, allowing the original low-resolution image to be directly input into the network, rather than requiring enlargement through the bicubic method as in SRCNN. Second, it modifies the feature dimensions, uses smaller convolutional kernels, and employs more mapping layers. Third, it allows for the sharing of mapping layers; models trained for different upsampling rates can simply fine tune the final deconvolution layer.

Methods like SRCNN require low-resolution images to be upscaled via interpolation, to match the size of high-resolution images before being input into the model. This necessitates performing convolution operations at a higher resolution, thereby increasing the computational complexity. Shi et al. [26] proposed an efficient method that extracts features directly at the low-resolution image size and computes the high-resolution image. The core concept of ESPCN is the sub-pixel convolutional layer. The input into the model is the original low-resolution image, which, after passing through three convolutional layers, produces a feature map with the same size as the input image but with a channel count of $r^{2}$. These features are then rearranged via the sub-pixel convolutional layer to form a high-resolution image.

The DRCN [9] was the first to apply an existing recursive neural network structure to super-resolution processing. It also utilized the concept of residual learning to deepen the network, which enlarged the layers’ receptive field and improved the performance. The authors of the DRRN [10] utilized a deeper network structure to achieve performance enhancements. In the DRRN, each residual unit shares the same input, which is the output from the first convolutional layer in the recursive block. Each residual unit contains two convolutional layers. Within a recursive block, the convolutional layers at corresponding positions in each residual unit share parameters (represented by the light green or light red blocks in the diagram).

The model proposed by Tong is called SRDenseNet [27], which utilizes dense blocks as the basic structure and employs skip connections to combine low-level feature information with high-level feature information. Subsequently, image reconstruction is carried out through a deconvolution network, facilitating the transformation from low resolution to high resolution. Furthermore, it is also highlighted that the information contained in the features across different depth layers is complementary.

Xin et al. [28] designed a Bit Accumulation Mechanism (BAM), using a value accumulation scheme to approximate full-precision convolution, refining the quantization precision progressively along the direction of the model inference. They also proposed an efficient model architecture based on BAM, named the Binary Super-Resolution Network (BSRN), to reduce the computational complexity and parameters. In experiments, they implemented BAM into VDSR and SRResNet to demonstrate the effectiveness of their method and compared it with the BSRN.

## 3. Binary Neural Networks

Binary neural networks (BNNs) refer to neural networks that use only two values, {+1, −1}, to represent weights and activations. Compared to full-precision neural networks, BNNs can replace the 32-bit float multiplication and accumulation, typically used in convolution operations with a much simpler combination of XNOR and popcount operations. Significantly, this substitution saves memory and computational resources, facilitating the deployment of models by resource-constrained devices. However, due to the limited amount of information that binary values can represent, BNNs have historically exhibited much lower accuracy than full-precision models. Nevertheless, recent studies such as those on MeliusNet [29], IRNet [24], and ReActNet [30], have made substantial efforts to improve BNNs, achieving over 70% top-1 accuracy on the ImageNet dataset.

The concept of binary neural networks (BNNs) originated from BinaryConnect [18], and was proposed by Courbariaux et al. To address the issue of gradient propagation in binarized weights, the authors propose maintaining a set of real-valued weights during training and then using the sign function to obtain the binarized weights. The binarization can be given by:

(1) $$B_{w}=Sign(R_{w})$$

where $B_{w}$ and $R_{w}$ denote the binarized and real-valued weights, respectively.

To overcome the issue where the sign function is non-differentiable at zero and has a derivative of zero elsewhere, which hinders effective gradient propagation, the authors designed the Straight-Through Estimator (STE).

XNOR-Net [31] builds upon the original BNN framework by accounting for quantization errors and introduces the use of scaling factors. Each output channel direction of the real-valued weights is associated with a scaling factor to restore the information lost in binarized weights. Similarly, each pixel in the height and width direction of the activation is associated with a scaling factor to recover the information lost in the binarized activations. These scaling factors do not require learning; they can be directly determined by calculating the corresponding L-1 norm. This method does not compromise the efficiency of binary convolution operations. Experimental results show significant improvements over the original BNN and, for the first time, demonstrate the performance of BNNs on a large dataset like ImageNet. In convolution operations, XNOR-Net achieves a speed-up of up to 58 times and saves 32 times the memory compared to traditional methods.

Liu et al. [30] initially adopted the concept from Bi-Real Net [21], which involves incorporating shortcut layers into the original network, to modify MobileNetV1. Through extensive experimentation, they found that the performance of BNNs is particularly sensitive to changes in the distribution of activations, specifically noting that shifts in and the scaling of activations have a significant impact on BNN performance. Consequently, the authors considered that each layer’s activations might have an optimal offset and scaling value that would maximize the model’s overall performance. This led to the proposal to modify the sign and PReLU functions to include learnable parameter variables, allowing the model to automatically learn the best offset and scaling values for each layer. These were named the ReAct-Sign (abbreviated as RSign) and ReAct-PReLU (abbreviated as RPReLU).

## 4. Neural Architecture Search

The architecture of a neural network, a critical component of deep learning models, plays a pivotal role in determining model performance. For instance, ResNet [19] (residual network) has greatly advanced image processing technology by addressing the issue of degradation during deep network training through the introduction of residual connections. However, as models become increasingly complicated, the performance enhancement also demands precise adjustments of the hyperparameters. Minor variations in the hyperparameters can significantly impact model performance, making experimental results difficult to replicate. This not only adds to the burden of researchers, but also raises the barrier to entry into the field. So, just as steam engines were gradually replaced by electric motors, the design of neural network architecture is transitioning from manual to automated design by machines. A landmark event in this process occurred in 2016, when Zoph and Le [15] utilized reinforcement learning for neural architecture search (NAS) and achieved superior performance on image classification and language modelling tasks compared to previous manually designed networks. This development highlights a significant shift towards automating the design process in the field of neural networks, promising enhancements in the efficiency and effectiveness of model architectures. With the development of NAS, automatic design using the NAS method for specific tasks has begun to outperform the finest architectures designed by humans in several domains, such as object detection [32,33], semantic segmentation [34,35], protein folding [36,37], and weather prediction.

As previously mentioned, network architectures can relieve designers of the cumbersome process of manual design. Particularly, the recently introduced gradient-based methods, such as DARTS [13], have enabled efficient searches for neural network architectures within a continuous search space. DARTS models neural architecture search (NAS) as a bi-level optimization problem, employing alternate optimization through gradient descent to derive the optimal network architecture.

**Figure 1:**
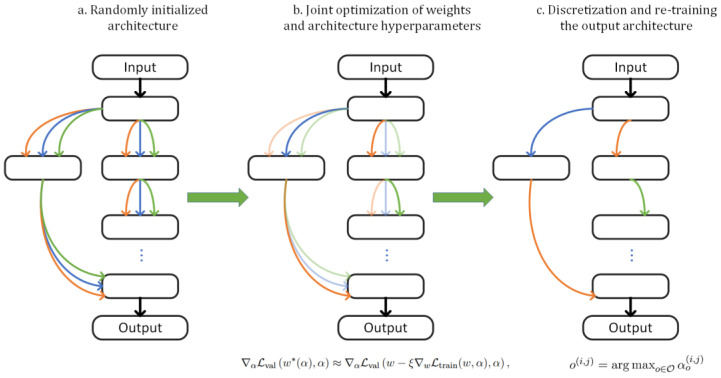
DARTS flow.

DARTS is also categorized under one-shot NAS methods, which involve constructing a super net and then extracting the best sub-network from it. This approach addresses the traditional black box nature of network structure searches, making them more transparent and detailed. The key lies in its use of soft functions to mix candidate operations. The gradient optimization approach pioneered by DARTS demonstrates remarkable results, evident from the minimal GPU days required to see outcomes [14]. This method stands out from other early NAS techniques [15,16,17], particularly those based on reinforcement learning or evolutionary algorithms, because it is not constrained by the intrinsic discrete optimization nature of these methods. Fundamentally, it addresses the issue that previous NAS methods required extensive computational resources. Figure 1 presents the flow of DARTS.

Since the introduction of DARTS, numerous researchers have proposed improvements, aiming to achieve better network architectures. In P-DARTS [38], researchers proposed a progressive approach to alleviate the depth gap in DARTS, and they also improved the stability of the architectures derived from the search by implementing skip-connect regularization. PC-DARTS [39] aims to significantly reduce the computational requirements and memory usage, allowing for faster searches with larger batch sizes. The authors designed a channel-based sampling mechanism, where only a small fraction of 1/K of the channels in a node are used for the operation search, reducing the memory usage by (K − 1)/K, thereby enabling an increase in the batch size by a factor of K. To address the instability caused by channel sampling, they introduced edge normalization, which reduces uncertainty during the search by learning the edge-level hyperparameters of the super net. Xue and Qin introduced a method called ADARTS [40], a differentiable neural architecture search based on channel attention, which utilizes partial channel connections. By using an attention mechanism, it selects channels with higher importance to be sent to the operation space, whereas the rest of the channels are directly connected with the processed channels. This approach has been shown to greatly improve search efficiency and memory usage, and to reduce instability in network structures that typically arises from random channel selection.

Courbariaux proposed a method using binary values to represent weights and activations in neural networks, which was later defined as the first binary neural network. In Courbariaux’s approach, through (1), full-precision weights and activation values are converted to binary values {−1, 1}, significantly reducing the hardware burden. Subsequently, multiplication operations within the model can be replaced with XNOR-popcount operations, which are described as follows:

(2) $$X*W\approx sign(X)\;⊙\;sign(W)=B_{x}\;⊙\;B_{w}$$

where X and W denote inputs and weights of the convolutional layer, respectively. And ∗ represents the convolution operation. The XNOR-popcount operation is denoted by ⊙.

A crucial consideration is that while binary neural networks (BNNs) offer advantages in terms of storage and computation speed, it must be acknowledged that model performance inevitably declines due to insufficient information representation of binary values. This issue can be mitigated through well-designed network architectures. However, to ascertain whether a structure can enhance neural network performance, extensive experimentation is required. Hence, the relevance of binary network architecture search techniques. The BNAS model [41] introduced a new binary search space and cell template, rediscovered the utility of the Zeroise layer, and implemented diversity regularizers to search for binary structures with improved performance. In BATS [42], in addition to designing a new binary-oriented search space, it also introduced a softmax with a temperature coefficient to foster more discriminative NAS.

## 5. Method

**Figure 2:**
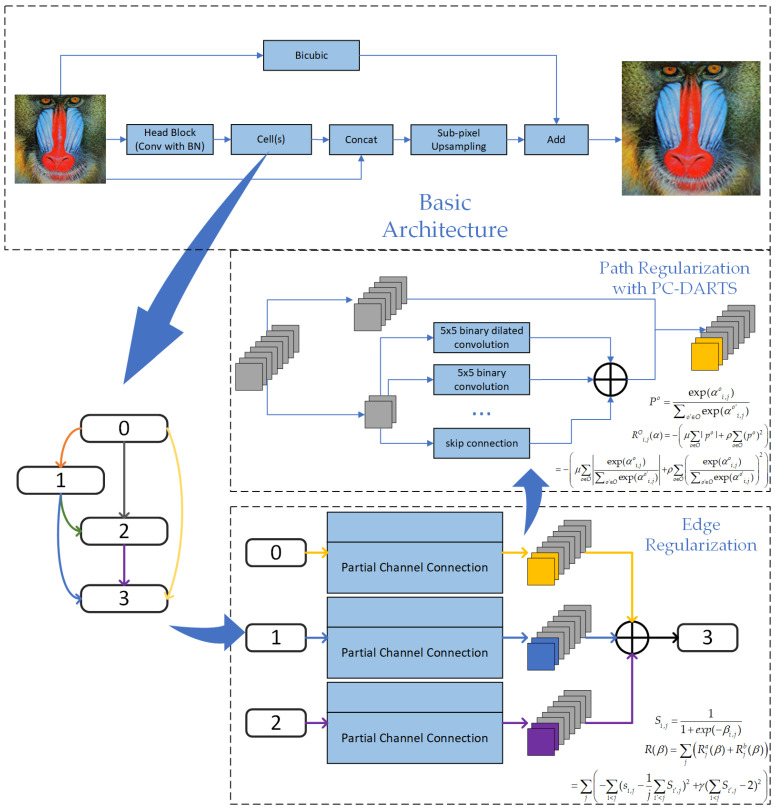
Search framework and architecture.

In the overall network framework and the basic structure of cells, we have made specific adjustments for super-resolution tasks to ensure the model’s suitability for such applications. A basic architecture is shown in Figure 2.

The fundamental concept of DARTS is to learn the model structure within a differentiable search space, seeking the best combination of operations by optimizing a parameterized search space. Within DARTS, operation selection is accomplished via the softmax function, which converts operation weights into a probability distribution. The operation selection can be expressed as:

(3) $$P^{o}=\frac{\exp({\alpha^{o}}_{i,j})}{\sum_{o^{\prime }\in O}\exp({\alpha^{o^{\prime }}}_{i,j})}$$

where ${\alpha^{o}}_{i,j}$ is the architecture parameter associated with the weight operation o. $P^{o}$ denotes the probability of operation o, which is based on the operation weights ${\alpha^{o}}_{i,j}$ calculated by the softmax function.

In DARTS, the architectural parameters associated with different operations exhibit minimal changes, and even slight fluctuations can induce alterations in the cells. Therefore, in our framework, we introduce L1 and L2 regularization to enhance the robustness of the entire search framework. The regularization term can be expressed as:

(4) $$\begin{array}{l} {R^{O}}_{i,j}(\alpha )=-\left(\mu \sum_{o\in O}\left|p^{o}\right|+\rho \sum_{o\in O}{\left(p^{o}\right)}^{2}\right)\\ =-\left(\mu \sum_{o\in O}\left|\frac{\exp({\alpha^{o}}_{i,j})}{\sum_{o^{\prime }\in O}\exp({\alpha^{o^{\prime }}}_{i,j})}\right|+\rho {\sum_{o\in O}\left(\frac{\exp({\alpha^{o}}_{i,j})}{\sum_{o^{\prime }\in O}\exp({\alpha^{o^{\prime }}}_{i,j})}\right)}^{2}\right) \end{array}$$

For L1 regularization in operation selection, it can be used to encourage the search framework to produce sparse operation selections, thereby achieving more efficient and stable operation selection. By adding the L1 regularization term to the loss function, the weights of some unimportant operations tend toward zero, thereby reducing the model complexity and improving the generalization ability. But applying only L1 regularization may result in the weight of some operations becoming very close to zero, which means that these operations are ignored during the search process, eventually leading to skip connect for most operations. Hence, we also introduce L2 regularization with balancing factor ρ to avoid the negative effect of L1 regularization. By controlling the strength of L2 regularization, one can effectively manage the complexity of the model and help it generalize better to unseen data. This method can help stabilize the search process, preventing search instabilities and, thus, better enabling the search for suitable model structures. The search object with regularization is given by:

(5) $$L(\theta ,\alpha )=L^{l1}(\theta ,\alpha )+\lambda \;Linear(R(\alpha ))$$

where $L^{l1}$ is the search object for the super-resolution task, which is an L1 loss. L1 loss is popular for SR tasks [43,44]. Where λ is a balancing factor. The introduction of linear interpolation is to avoid adding strong regularization in the early stages of the architecture search, which would cause the normalized weights of dominant operations to be further enlarged, making it difficult for other operations to stand out, even if they have better performance, in the later search stage. Hence, it is necessary to gradually increase the impact of regularization through linear interpolation, with the formula being:

(6) $$Linear=\frac{e}{E}$$

where *e* is the current epoch and *E* presents the expected epoch that has the maximum effect on the regularization. To further enhance the stability of the architecture search process, we also introduce edge normalization to balance the importance of each edge. This normalization method adopts the variance-based edge regularization in EBNAS [45]. The regularization is given by:

(7) $$\begin{array}{l} R(\beta )=\sum_{j}\left(R_{j}^{a}(\beta )+R_{j}^{b}(\beta )\right)\\ =\sum_{j}\left(-\sum_{i<j}{\left(s_{i,j}-\frac{1}{j}\sum_{i\prime <j}S_{i^{\prime },j}\right)}^{2}+\gamma {\left(\sum_{i<j}S_{i^{\prime },j}-2\right)}^{2}\right) \end{array}$$

Finally, the object with two instances of regularization can be expressed as:

(8) $$L(\theta ,\alpha )=L^{l1}(\theta ,\alpha )+\lambda \;Linear(R(\alpha ))+\mu \;Linear(R(\beta ))$$

Regarding search spaces, maxpool and avgpool operations contribute to learning positional invariance in image classification tasks, effectively recognizing features even if they shift or distort slightly in the image. This effectiveness is due to pooling operations that select certain values to reduce the spatial dimensions, thus deriving more robust representations of the features. However, in super-resolution tasks, using maxpool and avgpool is generally not recommended, as these pooling techniques can lead to some information loss during processing. The essence of super-resolution tasks lies in extracting high-resolution details from low-resolution images, which requires preserving as much information as possible, rather than reducing it. Hence, removing all of the pooling operations is necessary to avoid the model suffering from a large amount of information loss. Additionally, larger convolutional kernels can cover a broader area, capturing more extensive contextual information. In super-resolution tasks, a larger receptive field helps the network understand the relationships between pixels over a greater area, which is crucial for reconstructing high-quality, high-resolution images. This is the reason we maintained the dilated group convolution operation and introduced a 7 × 7 binary group convolution. Table 1 presents our search space.

**Figure 3:**
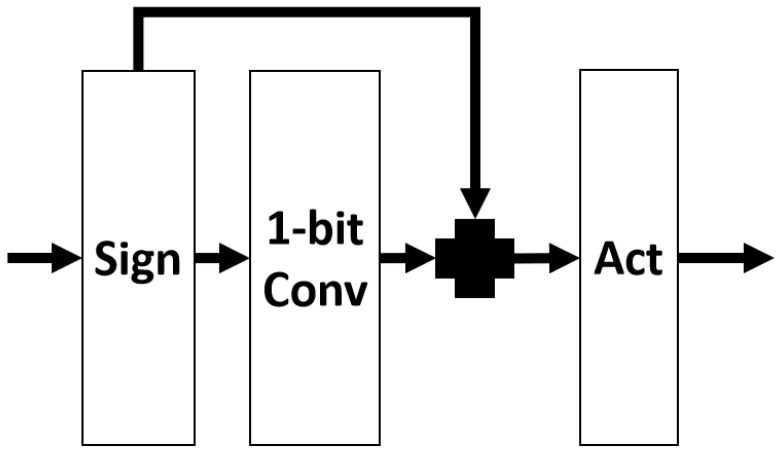
Basic structure of the operation in the search space.

In the search space, we made detailed structural modifications to each operation to adapt them for super-resolution tasks. For image super resolution, the model’s output image should match the input in terms of color, contrast, and brightness. The changes are primarily made in regard to the resolution and some details. Batch normalization (batch norm) acts as a contrast stretcher for images. When an image undergoes batch norm, its color distribution is normalized, which causes a loss of the original contrast information of the image. Therefore, batch norm actually impacts the quality of the model’s output. Figure 3 shows the basic structure of this operation in the search space.

**Figure 4:**
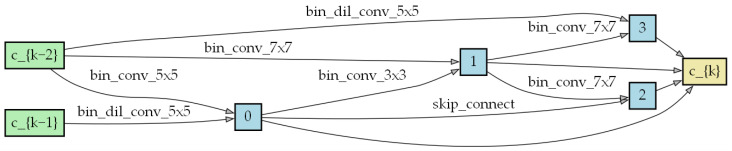
Example of a cell.

A cell consists of several different candidate operations in the search space. The Figure 4 shows an example of a cell, with each edge representing an operation.

The performance degradation in binary neural networks is primarily due to their limited representational capacity and the discrete nature of binarization, which leads to significant information loss during both forward and backward propagation. In forward propagation, when activations and weights are restricted to just two values, the diversity of the model sharply decreases. During backward propagation, accurate gradients are essential for providing correct optimization directions; however, binary networks often produce inaccurate gradients and incorrect optimization directions during training due to their discrete binary values. To address this problem, we introduce the quantization method from IR-Net [24].

During forward propagation, quantization operations lead to information loss. In many binarized convolutional neural networks, minimizing the quantization error is adopted as the objective function for optimization. The quantization can be formulated as

(9) $$Q_{x}(x)=B_{x}$$

where $B_{x}$ is the binarized activation or inputs according to the sign function. The objective function is given by:

(10) $$\min\;J(Q_{x}(x))={\left\|x-Q_{x}(x)\right\|}^{2}$$

The information entropy is performed for the binarized result $Q_{x}(x)$, which is in fact the information entropy of $B_{x}$, which follows a Bernoulli distribution. Thus, the formula can be expressed as:

(11) $$f(B_{x})=\left\{\begin{array}{ll} p, & ifB_{x}=+1\\ 1-p, & ifB_{x}=-1 \end{array}\right.$$

(12) $$H(Q_{x}(x))=H(B_{x})=-p\ln(p)-(1-p)\ln(1-p)$$

Moreover, the information entropy reaches its maximum when p=0.5, indicating that the quantized values are uniformly distributed. The objective function of Libra-PB is defined as follows:

(13) $$\min\;J(Q_{x}(x))-\lambda H(Q_{x}(x))$$

Additionally, to ensure more stable training and mitigate adverse effects caused by weights and gradients, further normalization was applied to balance the weights as follows:

(14) $${\hat{W}}_{std}=\frac{\hat{W}}{\sigma (\hat{W})},\;\;\;\hat{W}=W-\bar{W}$$

where $\bar{W}$ is the mean of the weight and σ(🞄) denotes the standard deviation. Through Equation (6), we gain the maximum information entropy of the weights, which makes the full-precision weight involved in binarization more spread out.

Moreover, in order to bypass expensive floating-point operations, while boosting the representational capability of binary weights, this approach incorporates integer scaling factors instead of floating-point ones. This adjustment allows binary calculations involving scaling factors to be simplified to:

(15) $$\begin{array}{c} Q_{w}({\hat{W}}_{std})=B_{W}<<>>s=sign({\hat{W}}_{std})<<>>S\\ S^{*}=round({log}_{2}(||W_{std}{\left|\right|}_{1}/n)) \end{array}$$

where <<>> denotes the left or right bit-shift operation. Finally, the binary convolution operation can be expressed as follow:

(16) $$Z=(B_{w}⊙B_{a})<<>>S$$

Due to the discontinuous nature of binarization, gradient approximation is an inevitable aspect of backward propagation. This makes it challenging to accurately model the effects of quantization, leading to significant information loss. To preserve the information derived from the loss function during backward propagation, we introduced a progressive two-stage approximation gradient method using EDE. In the first stage, we maintain the updating capability of the backward propagation algorithm by keeping the derivative values of the gradient estimation function close to 1, then gradually reducing the truncation value from a large number to 1. This rule allows our approximation function to evolve from an identity function to a clip function, ensuring early training updates. In the second stage, we keep the truncation value at 1 and gradually evolve the derivative curve to the shape of a step function. Using this rule, our approximation function transitions from a clip function to a sign function, thus ensuring consistency between forward and backward propagation.

Of course, in addition to the improvements above, the basic architecture of DARTS is also unsuitable for SR tasks. Therefore, for the application of a binary neural network in SR, we propose a hierarchical basic architecture, as shown at the top of Figure 2. Through two full-precision shortcuts, we attempt to pass image information from different stages as much as possible without adding a large amount of computation, in order to further compensate for the information loss caused by binary neural networks.

## 6. Experiments

In terms of model training, to ensure the fairness of ablation studies, all models are trained on the publicly available Timofte dataset [46], which comprises 91 images designed for training purposes. For testing, the Set5 [47] and Set14 [48] datasets are used, containing 5 and 14 images, respectively. Additionally, the Berkeley Segmentation Dataset, consisting of 100 images (BSD100), is utilized for model evaluation. The training and testing were performed on the RTX4080 GPU. In the experiments, we configured each search to have 100 epochs, with a search framework learning rate of 0.0006. Upon completion of the search, model training was conducted using a learning rate of 0.001.

To simplify the comparison, we adopted the parameters from EBNAS for the balancing factors λ and γ, which are 1.5 and 0.8, respectively. The experiments primarily focused on the impact of the balancing factors in the alpha regularization term. We conducted three sets of experiments in which the balancing factors μ and ρ are 0.2 and 0.8, 0.5 and 0.5, and 0.8 and 0.2, respectively.

**Figure 5:**
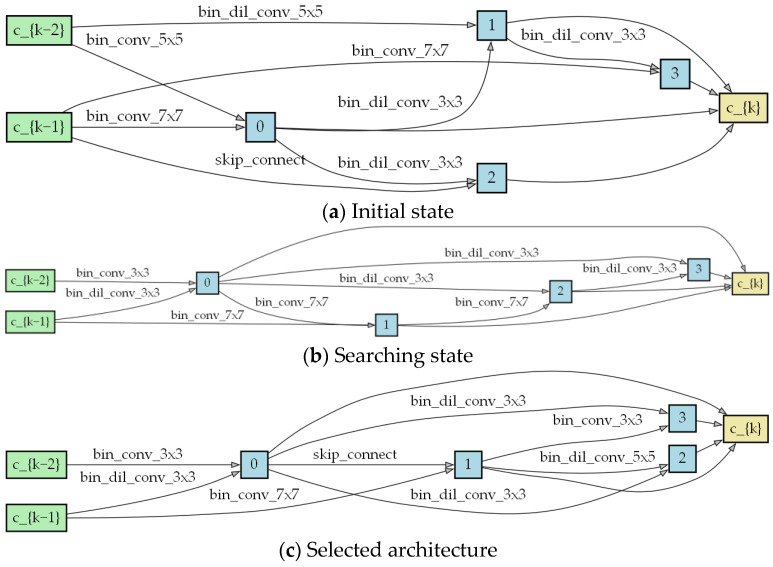
Cell structure, with scaling factor 3, after the search process.

**Figure 6:**
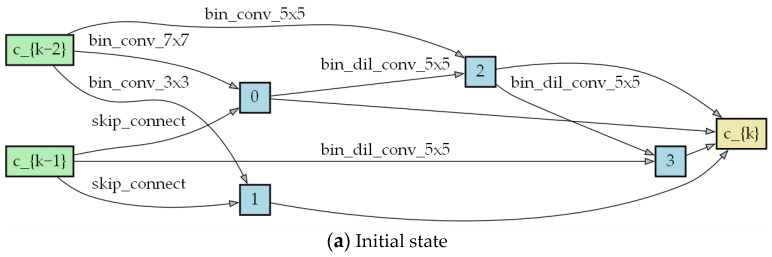

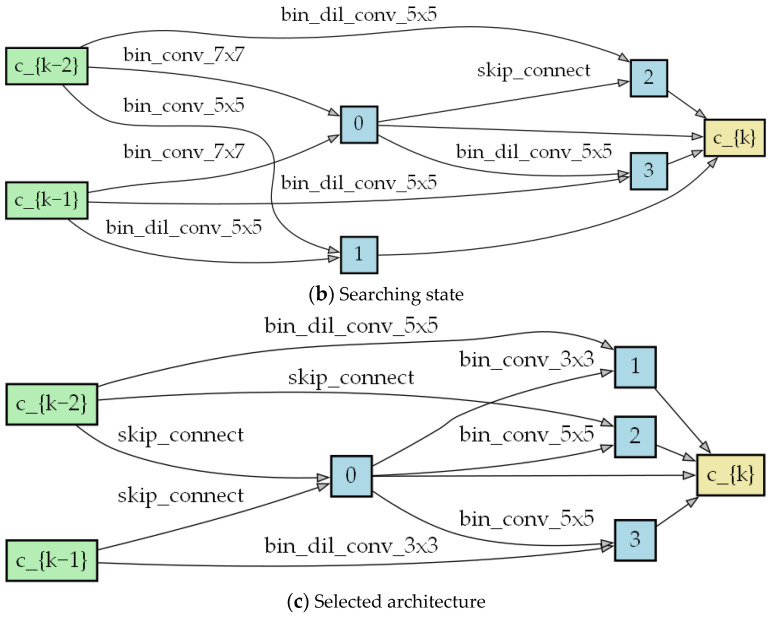
Cell structure, with scaling factor 4, after the search process.

We trained a lightweight network comprised of three cells and a larger network consisting of eight cells, with scaling factors equal to 3 and 4. For the three-cell lightweight network, each layer has 64 channels, and all parameters are optimized using Adam with a channel sampling factor of k = 8. This model was trained over 100 epochs, with a batch size of 256. Figure 5 and Figure 6 show the cell structure after the search process.

For the evaluation, the peak signal-to-noise ratio (PSNR) is a widely used metric in super-resolution tasks, used to evaluate the quality of high-resolution images obtained through models compared to ground truth images. It measures the similarity between two images, while considering the pixel values and image size.

The structural similarity (SSIM) metric is a metric used to measure the degree of similarity between two images and is commonly used in image quality evaluation.

Table 2 presents the performance comparison using the manual method on the three datasets, where our SRBNAS represents our 3-cell model searched by super net. When compared with manually designed models, the models derived from our network architecture search method show performance that rivals those based on the three evaluation datasets. The table shows that the SRBNAS results have very good performance. The results are shown for scaling factors 3 and 4.

Additionally, as depicted in Table 3, it becomes apparent that the model structures used are particularly efficient. Our SRBNAS model requires only about 156 K number of parameters to achieve an image reconstruction performance comparable to other methods. These results clearly demonstrate the high efficiency of our method. Compared to manual design approaches, NAS can save a significant amount of trial-and-error design time.

First, in Table 4, μ represents the balancing factor of L1 regularization, while ρ represents the balancing factor of L2 regularization. From the results of the PSNR, there is no significant difference in the performance of the model structures obtained from the search. However, as the regularization weight tends toward the L1 regularization term, the model size begins to drop sharply. Yet, in terms of model performance, the decrease in model size makes the model more efficient and lightweight. Specifically, L1 regularization tends to produce sparse weights, i.e., to make some weights tend toward zero, thus reducing the complexity of the model, while L2 regularization makes the weight distribution smoother and avoids the weights being too large or too small. Therefore, combining these two types of regularization and adjusting the weights of the L1 regularization appropriately can achieve the effect of simplifying the model and reducing the amount of computation.

**Figure 7:**
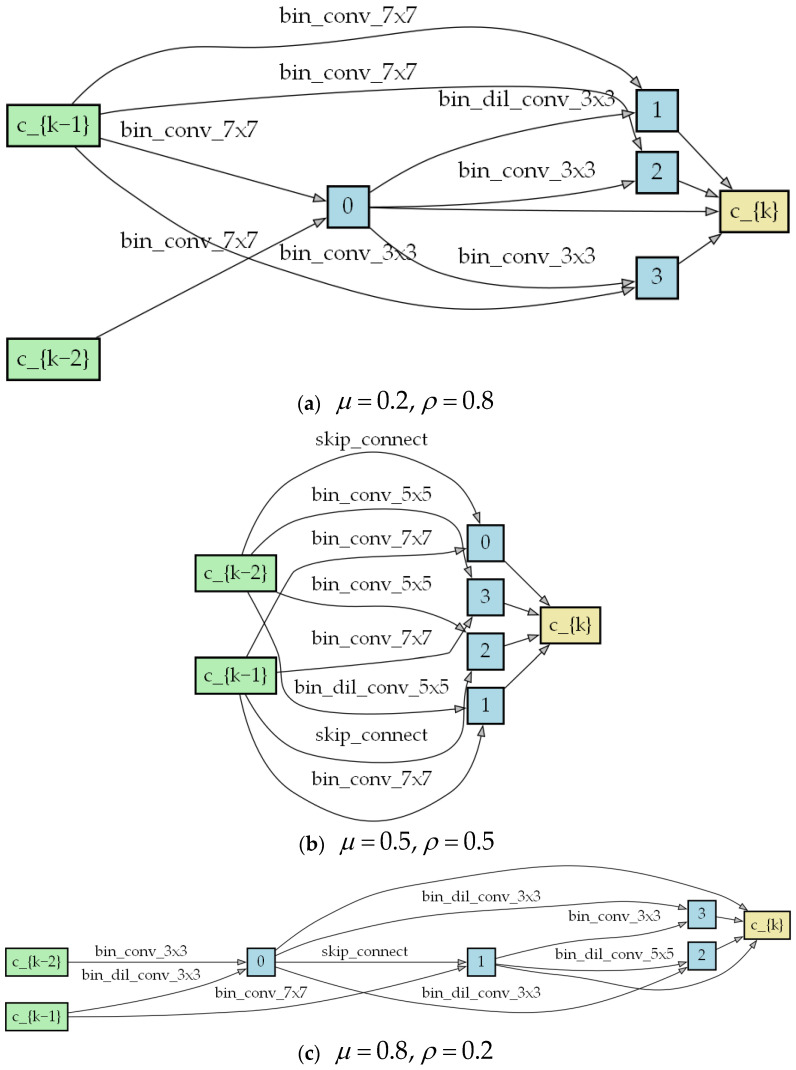
Cell structure with different combinations of regularization.

Figure 7 shows the cell structure results according to the different combinations of balancing factors. From the figure, as ρ increases, high-computation operations in the model also increase accordingly, such as 5 × 5 and 7 × 7 convolutions and a lack of skip connections. The number of parameters increases significantly, but there is not much improvement in terms of the performance. However, as μ increases, the operations in the cell structure are more of a lower computational complexity, such as 3 × 3 and 5 × 5 dilation convolutions, or 3 × 3 convolutions.

After a comparison with the manual method, for a fair comparison Table 5 presents the computational comparison with other gradient-based architecture search method for super resolution. The table shows the reduced computational requirements for our SRBNAS method compared with two other methods (DLSR [48] and DNAS-EASR [49]).

**Figure 8:**
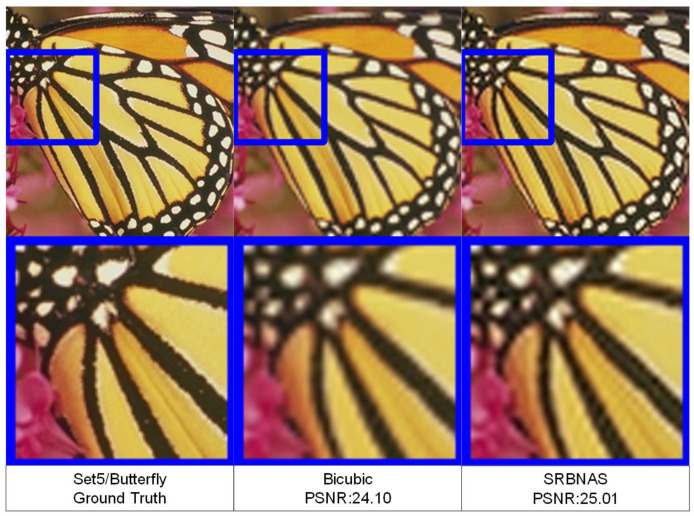
Visual quality comparisons of a HR image of a butterfly from Set5, with an upscaling factor of 3.

**Figure 9:**
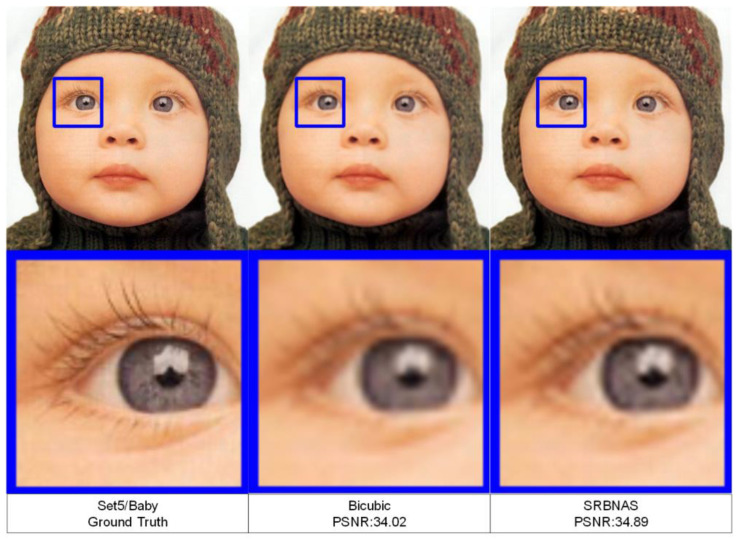
Visual quality comparisons of a HR image of a baby from Set14, with an upscaling factor of 3.

To visualize the model performance, Figure 8 and Figure 9 display three different HR images. We also zoomed in on the images to facilitate the observation of changes in the image details.

## 7. Conclusions

Based on binary neural networks (BNNs), this paper proposes a network architecture search method called SRBNAS, specifically for super-resolution tasks. The method utilizes several techniques to achieve performance and computational improvements. By integrating Libra Parameter Binarization (Libra-PB), the method aims to preserve as much information as possible during forward propagation. The inclusion of an Error Decay Estimator (EDE) during backward propagation assists in effectively handling the reduction in the parameter update capabilities outside the truncation range and mitigating information loss due to approximate errors within that range. These strategies effectively lessen the discrepancies between the search phase and the evaluation phase. The experimental results demonstrate that the network structures identified by this search method have a good level of performance and are computationally efficient compared to other approaches and manually designed alternatives.

## 8. Patents

This section is not mandatory but may be added if there are patents resulting from the work reported in this manuscript.

## Figures and Tables

**Table 1:** Search space for BNNs in regard to SR.

3 × 3 binary dilated group convolution
3 × 3 binary group convolution
5 × 5 binary dilated group convolution
5 × 5 binary group convolution
7 × 7 binary group convolution
skip connection

**Table 2:** The mean PSNR/SSIM of different methods evaluated using different datasets.

Dataset	Scale	Bicubic	ESPCN (Full Precision)	ResBinESPCN-A2 [49]	SRResNet (BNN)	VDSR (BNN)	Ours: SRBNAS
Set5	×3	30.46/0.87	32.29/-	29.82/-	31.18/0.88	31.01/0.87	31.80/0.90
Set14		27.59/0.78	28.90/-	27.33/-	28.29/0.80	28.15/0.79	28.57/0.81
BSDS100		27.26/0.75	28.16/-	27.03/-	27.73/0.77	27.57/0.76	27.95/0.78
Set5	×4	28.48/0.82	28.80/-	28.11/-	29.33/0.82	29.02/0.82	29.34/0.84
Set14		25.92/0.72	26.16/-	25.78/-	26.72/0.72	26.55/0.72	26.47/0.74
BSDS100		26.02/0.67	26.21/-	25.87/-	26.45/0.69	26.29/0.69	26.45/0.70

**Table 3:** Number of parameters for each model with a scale factor of 3.

Model	Parameters
VDSR_BAM	668 K
SRResNet BAM	1547 K
BSRN	1216 K
ResBinESPCN-A2	349 K
Ours: SRBNAS	192 K

**Table 4:** A comparison of different balancing factors with a scaling factor of 3.

Factor	Parameters	PSNR
μ=0.2, ρ=0.8	366 K	32.17
μ=0.5, ρ=0.5	350 K	32.02
μ=0.8, ρ=0.2	192 K	31.84

**Table 5:** Computational comparison with other gradient-based architecture search methods for SR.

Method	Scale	Param	Set5	Set14	BSDS100	Search Cost
DLSR [50]	×4	338 K	32.33	27.85	27.61	2 GPU days (RTX3080)
DNAS-EASR [51]		555 K	32.18	28.64	27.61	21 GPU hours (RTX3090)
Ours SRBNAS		156 K	29.34	26.47	26.45	1.2121 GPU hours (RTX4080)

## Data Availability

The original contributions presented in the study are included in the article, further inquiries can be directed to the corresponding author/s.
